# Palmitic Acid Induces Production of Proinflammatory Cytokines Interleukin-6, Interleukin-1**β**, and Tumor Necrosis Factor-**α** via a NF-**κ**B-Dependent Mechanism in HaCaT Keratinocytes

**DOI:** 10.1155/2013/530429

**Published:** 2013-08-29

**Authors:** Bing-rong Zhou, Jia-an Zhang, Qian Zhang, Felicia Permatasari, Yang Xu, Di Wu, Zhi-qiang Yin, Dan Luo

**Affiliations:** Department of Dermatology, The First Affiliated Hospital of Nanjing Medical University, Nanjing 210029, China

## Abstract

To investigate whether palmitic acid can be responsible for the induction of inflammatory processes, HaCaT keratinocytes were treated with palmitic acid at pathophysiologically relevant concentrations. Secretion levels of interleukin-6 (IL-6), tumor necrosis factor-**α** (TNF-**α**), interleukin-1**β** (IL-1**β**), NF-**κ**B nuclear translocation, NF-**κ**B activation, Stat3 phosphorylation, and peroxisome proliferator-activated receptor alpha (PPAR**α**) mRNA and protein levels, as well as the cell proliferation ability were measured at the end of the treatment and after 24 hours of recovery. Pyrrolidine dithiocarbamate (PDTC, a selective chemical inhibitor of NF-**κ**B) and goat anti-human IL-6 polyclonal neutralizing antibody were used to inhibit NF-**κ**B activation and IL-6 production, respectively. Our results showed that palmitic acid induced an upregulation of IL-6, TNF-**α**, IL-1**β** secretions, accompanied by NF-**κ**B nuclear translocation and activation. Moreover, the effect of palmitic acid was accompanied by PPAR**α** activation and Stat3 phosphorylation. Palmitic acid-induced IL-6, TNF-**α**, IL-1**β** productions were attenuated by NF-**κ**B inhibitor PDTC. Palmitic acid was administered in amounts able to elicit significant hyperproliferation and can be attenuated by IL-6 blockage. These data demonstrate for the first time that palmitic acid can stimulate IL-6, TNF-**α**, IL-1**β** productions in HaCaT keratinocytes and cell proliferation, thereby potentially contributing to acne inflammation and pilosebaceous duct hyperkeratinization.

## 1. Introduction

Acne is a chronic inflammation of the pilosebaceous units in certain area, including face and trunk, that mainly occurs in adolescence. Its pathogenesis is complex and is dependent on the interplay of multiple factors such as genetic predisposition, excess of sebum production, abnormal follicular proliferation, and development of inflammation [1]. Inflammation is indicated as a key component of the pathogenesis of acne [2]. An immunological reaction to the gram-positive microbe Propionibacterium acnes may play a major role in the initiation of the inflammatory reaction [3]. However, some published studies also indicate that in addition to Propionibacterium acnes, some components such as free fatty acid (FFA), arachidonic acid, linoleic acid, and some proinflammatory cytokines are associated with acne inflammation, and inflammation-inducing effects may not depend on the presence of Propionibacterium acnes. In addition, peroxisome proliferator-activated receptor (PPAR)*α* and neural factors are also related to acne inflammation [4–7]. 

Human sebaceous glands secrete a lipid mixture containing squalene and wax esters, as well as cholesterolesters, triglycerides, and possibly some free cholesterol [8–10]. Sebaceous lipids are responsible for the three-dimensional organization of skin surface lipids and the integrity of the skin barrier [11]. Besides, sebaceous lipids and its products were detected to express proinflammatory and anti-inflammatory properties [6, 12]. The early study found that FFA induces skin inflammation and stimulates sebaceous duct hyperkeratosis in animal models. Zouboulis evaluated the experimental results of patients with inflammatory lesions successfully treated with a new anti-inflammatory agent that specifically blocks the formation of leukotriene (LT) B4, demonstrating a significant reduction of FFA in sebum [12]. The decrease in FFA directly correlated with the improvement of inflammatory lesions. It is known that bacterial hydrolases convert some of the triglycerides to FFA on the skin surface [13]; however, there is also evidence indicating that sebaceous glands can also synthesize considerable amounts of FFA [12]. The main ingredients of FFA secreted by sebaceous glands are linoleic acid (LA), palmitic acid (PA), and oleic acid (OA). The composition of comedonal free fatty acids has been studied, demonstrating that the proportion of LA is markedly decreased in acne comedones, while PA is significantly increased [14, 15]. Akamatsu et al. have found that the decreased levels of LA in acne comedones contribute, in part, to the worsening of acne inflammation by the failure of low level of LA to inhibit neutrophil ROS generation and phagocytosis [16]. Further studies have indicated that PA can reduce the neutrophils to produce hydrogen peroxide; by their role in oxidative stress and damage to the epidermal barrier function, the proinflammatory mediators thereby more easily pass through the hair follicles into the dermis and aggravate acne inflammation [17]. However, the mechanisms of FFA in inducing acne inflammation have not been thoroughly studied. 

A number of proinflammatory cytokines, including interleukin-6 (IL-6), tumor necrosis factor-*α* (TNF-*α*), and interleukin-1*β* (IL-1*β*), have been implicated in the inflammatory process of acne [18, 19]. IL-6 has been shown to be a key player in acute and chronic inflammation [20]. Serum IL-6 levels were significantly higher in acne patients than that in normal population, suggesting a role for IL-6 in the pathogenesis of acne [21]. TNF-*α* and IL-1*β* can be induced by NF-*κ*B activation [22]. These two cytokines (TNF-*α* and IL-1*β*) propagate the acne inflammatory response by acting on endothelial cells to elaborate adhesion molecules to facilitate recruitment of inflammatory cells into the skin [23, 24]. 

The aim of this study was to investigate the possible role of PA in the initiation and development of inflammatory events using human HaCaT keratinocytes as model. We evaluated the effect of PA on IL-6, TNF-*α*, and IL-1*β* secretion in HaCaT cells. We also focused on the activation of NF-*κ*B, which coordinates the expression of different proinflammatory genes, secretion of the cytokine IL-6, TNF-*α*, and IL-1*β*, and induction of PPAR*α*. The latter inhibits the synthesis of proinflammatory molecules via a decreased activity of the NF-*κ*B signaling pathway [25]. 

## 2. Materials and Methods

### 2.1. Materials

Palmitic acid (PA) powder was bought from Sigma Co. Ltd. PA powder was added to a 10% solution of fatty acid free BSA and dissolved by shaking gently overnight at 37°C to yield an 200 mmol/L solution of PA complexed to BSA. Antibodies for PPAR-*α*, p-Stat3, and total-Stat3 were bought from Cell Signaling Technology, California, USA; antibody for LaminB was bought from Santa Cruz Biotechnology, CA, USA; antibodies for NF-*κ*B p65, IKK*α*, I*κ*B*α*, *β*-Actin, Cy3-conjugated mouse anti-rabbit immunoglobulins, pyrrolidine dithiocarbamate (PDTC, a selective chemical inhibitor of NF-*κ*B), cell culture supplies, CCK-8 Kit, and BCA Protein Assay Kit were all bought from Beyotime Institute of Biotechnology; Trizol was bought from Invitrogen, Carlsbad, CA; real-time PCR Assay Kits were bought from Nanjing KGI Bioteknologi Development Co, Ltd. IL-6 Sandwich ELISA Kit was bought from JingMei Bioengineer Company, Shenzhen. Helenalin was bought from Merlin Standard Chemicals, Singapore. Goat anti-human IL-6 polyclonal neutralizing antibody was obtained from R&D Systems, Minneapolis, MN, USA. 

### 2.2. Method

#### 2.2.1. Cell Culture

Keratinocyte line HaCaT cells were cultured in a cell incubator at 37°C, 5% CO_2_, in DMEM medium containing 10% fetal bovine serum and 1% penicillin and streptomycin. After cells became polygon arranging as a single layer, they were vaccinated at the density of 1 × 10^9^/L with 0.25% trypsin solution. The cultured cells were used for experiment when they adhered to the culture plate and the confluence reached 70%~80%.

#### 2.2.2. Experiment Grouping and Treatment of Cells

HaCaT keratinocytes were either without or with pretreatment for 1 hour with 10 *μ*M PDTC and then treated in serum-free conditions for 24 hours with PA at concentrations of 75, 100, 125, and 150 *μ*mol/L. For certain experiments, goat anti-human IL-6 polyclonal neutralizing antibody was added into cell culture system at a concentration of 10 *μ*g/mL. At the end of the treatment, fresh medium was added. Cells were collected at the end of the treatment or after 24 hours from the addition of fresh medium. As for IL-6, IL-1*β*, and TNF-*α* release detection, at the end of the treatment, supernatants were collected, fresh medium was added, and free cell supernatants were collected after 24 hours. 

#### 2.2.3. Immunofluorescence and Confocal Microscopy Detection of NF-*κ*B p65 in HaCaT Cells

The cells were washed with 0.01 M phosphate-buffered saline (PBS) and fixed in 4% formaldehyde for 30 min at room temperature. After being permeabilized with 1% Triton X-100 for 10 min, the cells were blocked with PBS containing 5% bovine serum albumin for 30 min at room temperature and immunofluorescent staining was performed using a specific mouse polyclonal antibody against NF-*κ*B p65 (dilution, 1 : 500) followed by Cy3-conjugated mouse anti-rabbit immunoglobulins (red). The slides were counterstained with Hoechst 33258 (blue). Finally, the cover slips were mounted on the slides and fluorescence was visualized using a confocal laser fluorescence microscope (Carl Zesis Zen 2008, Carl Zeiss Inc., Germany). Photographic images were taken from five random fields. Substitution of the primary antibody with a normal mouse IgG was used as control.

#### 2.2.4. Preparation of Cytoplasmic and Nuclear Extracts

Briefly, cells were scraped from dishes in PBS, pelleted, washed in hypotonic buffer (10 mM HEPES buffer, pH 7.9, 1.5 mM MgCl_2_, 5 mM KCl, 1 mM PMSF, 1 mM dithiothreitol, 1 mM Na_3_VO_4_, 1 mM NaF), and lysed by resuspension in the same buffer with 0.1% Nonidet P-40. Cytoplasmic extracts were isolated by centrifugation at 10,000 rpm for 10 min. Nuclear pellets were washed in hypotonic buffer and resuspended in cold extraction buffer (20 mM HEPES, 25% glycerol, 450 mM KCl, 1 mM EDTA, 1 mM PMSF, 1 mM Na_3_VO_4_, 1 mM NaF), gently agitated at 4°C for 45 min, and spun at 13,000 rpm for 30 min at 4°C. Supernatants were collected, and protein concentrations were determined with BCA Protein Assay Kit.

#### 2.2.5. Western Blotting Tests for the Protein Expression of NF-*κ*B p65, IKK*α*, I*κ*B*α*, p-Stat3, and PPAR-*α* in HaCaT Cells

An aliquot of protein extracted from cytoplasmic or nuclear extracts was subjected to 10% SDS-PAGE by electrophoresis under reducing conditions and transferred to PVDF membrane. The blotted membrane was then blocked with 5% nonfat dry milk in 1 × TBS (0.1% Tween 20) for 1 h at room temperature and incubated overnight at 4°C with primary antibodies to NF-*κ*B p65 (dilution, 1 : 500), to IKK*α* (dilution, 1 : 250), to I*κ*B*α* (dilution, 1 : 250), to PPAR-*α* (dilution, 1 : 500), to p-Stat3 (dilution, 1 : 500), to total-Stat3 (dilution, 1 : 500), to *β*-actin (dilution, 1 : 2,000), and to LaminB (dilution, 1 : 1,000). Following the incubation with horseradish peroxidase-conjugated sheep anti-mouse secondary antibodies (dilution, 1 : 2,000) for 1 h at room temperature, the blotted membrane was detected by Pierce ECL reagents (Thermo Fisher Scientific) and captured on X-ray film. Quantification of protein bands was established by Band-Scan software (PROZYME, San Leandro, California, USA) and expressed as a value relative to the density of the internal control (*β*-actin or LaminB).

#### 2.2.6. Real-Time PCR Detection of the Expression of PPAR-*α* mRNA in HaCaT Cells

Trizol was added to break down the cells, followed by extraction of total RNA, measurement of concentration, and then measurement of purity. After ensuring that the quality met the requirements of the experiment, cDNA was obtained by reverse transcription. It was diluted 10 times and amplified according to a 20 *μ*L reaction system. Primers were synthesized by Nanjing Kaiji Bio-tech Co., Ltd. (Table 1). Amplification conditions are as follows: pre-degeneration at 95°C for 5 min, entering reaction circles, degeneration at 95°C for 15 min, annealing for 30 s at 60°C, extending for 30 s at 72°C, keeping at 72°C for 10 min after 40 cycles.

#### 2.2.7. ELISA Analysis of the Expression of IL-6, IL-1*β*, and TNF-*α* in Cell Supernatants

Measurement of IL-6, IL-1*β*, and TNF-*α* were performed using commerical ELISA kits. This assay uses the quantitative sandwich immunoassay technique. The standard curve demonstrated a direct relationship between OD and secreted cytokine levels.

#### 2.2.8. Cell Proliferation Assay

Cell proliferation was assayed using a CCK-8 Kit. In brief, 100 *μ*L of cells (2 × 10^3^ cells/well) was transferred into 96-well plates after digestion with trypsin, and five parallel wells were used for each treatment. After attachment, the cells were subjected to the different treatments and then cultured for 24 h in a 5% CO_2_ incubator at 37°C. Subsequently, 10 *μ*L of CCK-8 was added to each well, and the cells were cultured for another 3 h. Cell density was determined by measuring the absorbance at 450 nm using a Varioskan Flash (Thermo Scientific, USA).

### 2.3. Statistical Analysis

SPSS13.0 software was used for data analysis, and the form of average ± standard deviation was used to indicate measurement data. ANOVA was used for intergroup comparison, *P* < 0.05 was considered statistically significant.

## 3. Results

### 3.1. Induction of IL-6, IL-1*β*, and TNF-*α* Secretion in HaCaT Keratinocytes by PA

**Figure 1:**
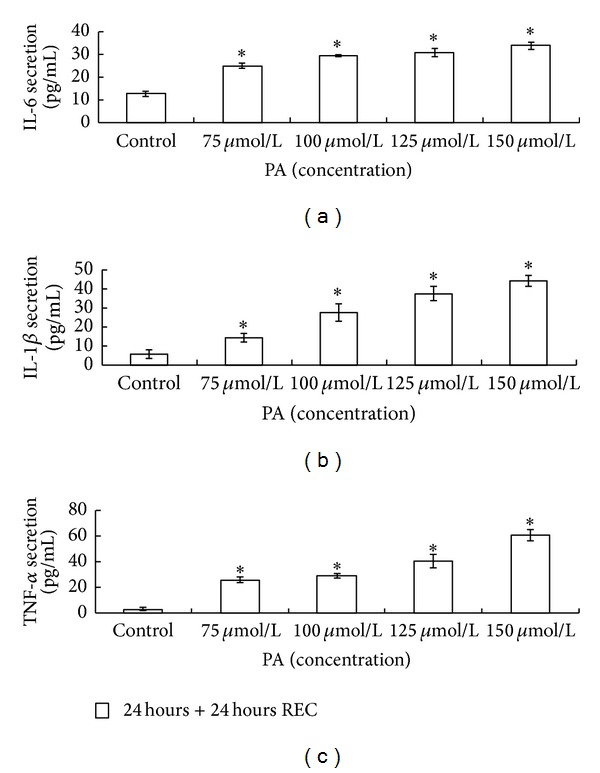
PA induces an upregulation of IL-6, IL-1*β*, and TNF-*α* secretion in HaCaT keratinocytes. HaCaT keratinocytes were untreated or treated with PA (75, 100, 125, and 150 *μ*mol/L) for 24 hours under serum-free conditions, and collected 24 hours after PA depletion. IL-6 (a), IL-1*β* (b), and TNF-*α* (c) releases were determined by ELISA kits. Results are expressed as average mean of protein concentration (pg/mL) ± SD and represent the mean of three experiments in duplicate. Asterisks (*) indicate significant differences of *P* < 0.05, respectively, between the PA-treated groups and nontreated group.

We observed, by means of ELISA analysis, an increase in a dose-dependent manner in the release of IL-6, IL-1*β*, and TNF-*α* in HaCaT keratinocyte supernatant treated with PA at a concentration of 75, 100, 125, and 150 *μ*mol/L 24 hours after PA removal (Figures 1(a), 1(b) and 1(c)). The obtained results demonstrate that PA is able to induce an inflammatory stimulus in HaCaT keratinocytes by increasing IL-6, IL-1*β*, and TNF-*α* secretion.

### 3.2. Induction of NF-*κ*B Nuclear Translocation in HaCaT Keratinocytes by PA

**Figure 2:**
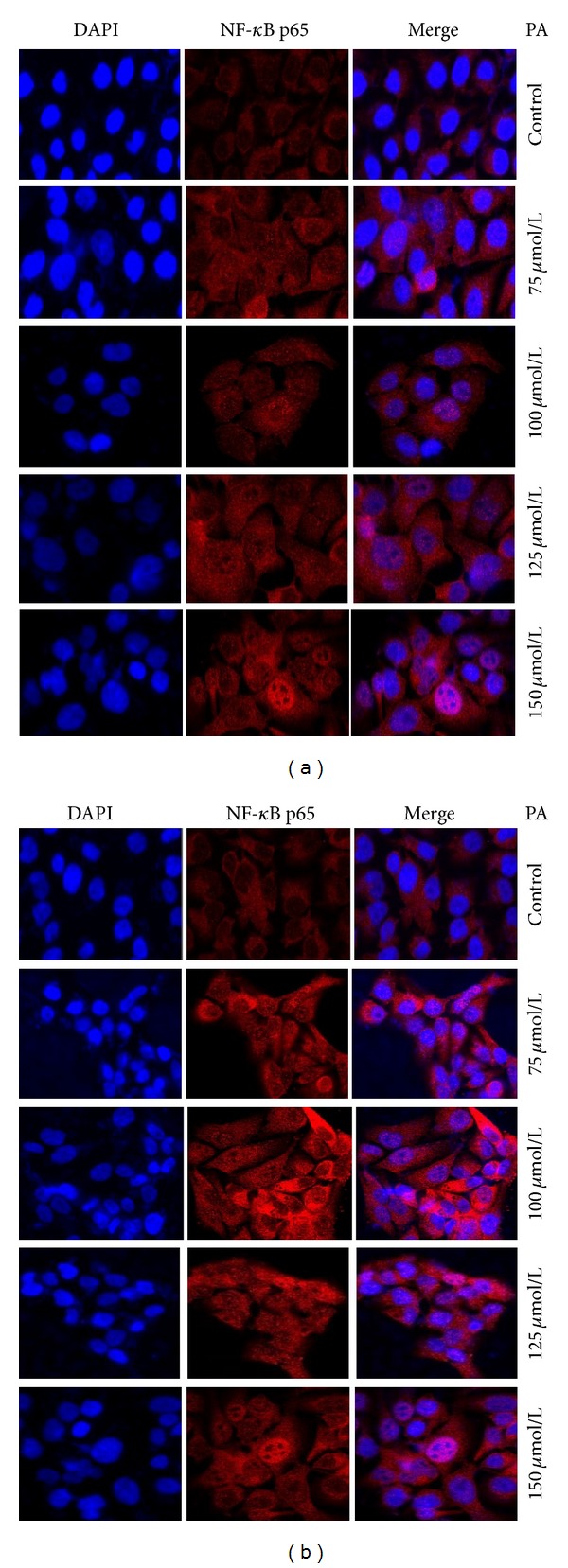
PA-induced nuclear translocation of NF-*κ*B p65 subunit in HaCaT cells immediately after the treatment and 24 hours after the PA depletion. Keratinocytes were untreated or treated with PA (75, 100, 125, and 150 *μ*mol/L) for 24 hours under serum-free conditions. Immunostaining was performed with specific mouse anti-p65 antibody followed by Cy3-conjugated mouse anti-rabbit immunoglobulins (red) immediately after the treatment (a) and 24 hours after the PA depletion (b). Images are representative of three independent experiments.

**Figure 3:**
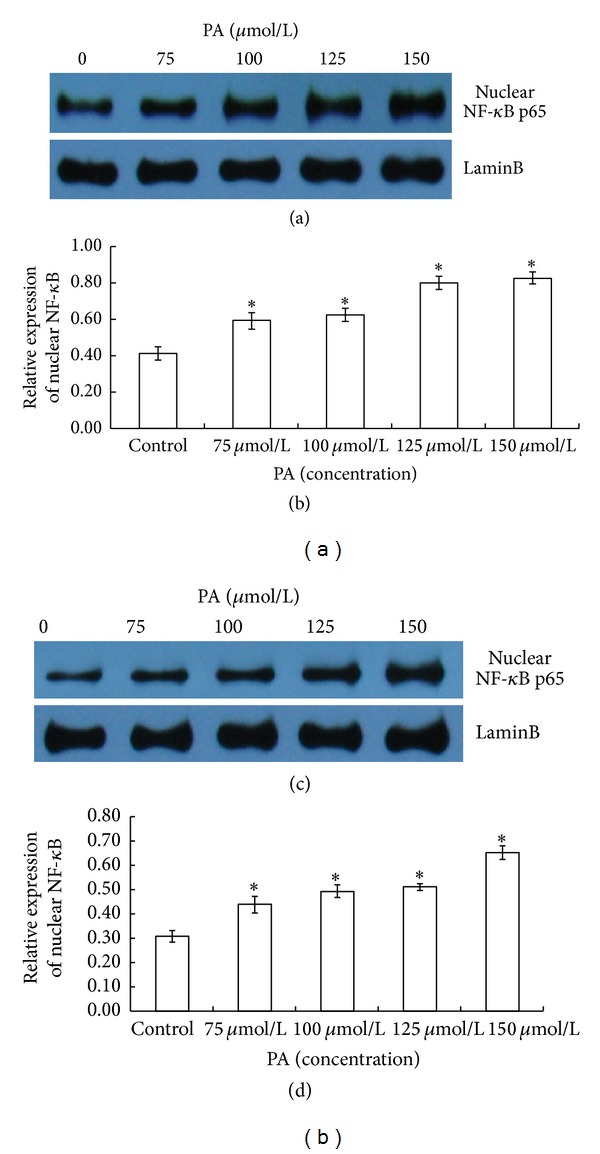
PA induces nuclear translocation of NF-*κ*B p65 subunit in HaCaT cells measured by western blotting. Keratinocytes were untreated or treated with PA (75, 100, 125, and 150 *μ*mol/L) for 24 hours under serum-free conditions. The cells were harvested, and nuclear extracts were analyzed with NF-*κ*B p65 by western blot immediately after the treatment (a) and 24 hours after the PA depletion (c). The densitometry values are means ± SD of three independent experiments ((b) and (d)). Asterisks (*) indicate significant differences of *P* < 0.05, respectively, between the PA-treated groups and nontreated group.

We used immunofluorescence staining to examine the localization of NF-*κ*B p65 in HaCaT keratinocytes. NF-*κ*B p65 was stained with Cy3-conjugated mouse anti-rabbit immunoglobulins (red) and nuclei were stained with Hoechst 33342 (blue). We found that NF-*κ*B p65 positive staining was predominantly localized in control cytoplasm (Figure 2). Interestingly, NF-*κ*B p65 staining significantly shifted to the nuclei with PA stimulation immediately after the treatment (Figure 2(a)) and 24 hours after the PA depletion (Figure 2(b)). The nuclear translocation of NF-*κ*B p65 subunit was further confirmed by the results derived from the western blotting studies. Upregulation of nuclear NF-*κ*B p65 protein levels in a dose-dependent manner was observed in cells treated with PA at 75, 100, 125, and 150 *μ*mol/L compared to control, immediately after the treatment (Figure 3(a)). The level of nuclear NF-*κ*B p65 protein expression in treated cells also increased dose dependently 24 hours after PA removal (Figure 3). The results obtained in these results demonstrate that PA is able to induce NF-*κ*B nuclear translocation in HaCaT keratinocytes. 

### 3.3. Induction of IKK*α* Activation and I*κ*B*α* Degradation in HaCaT Keratinocytes by PA

**Figure 4:**
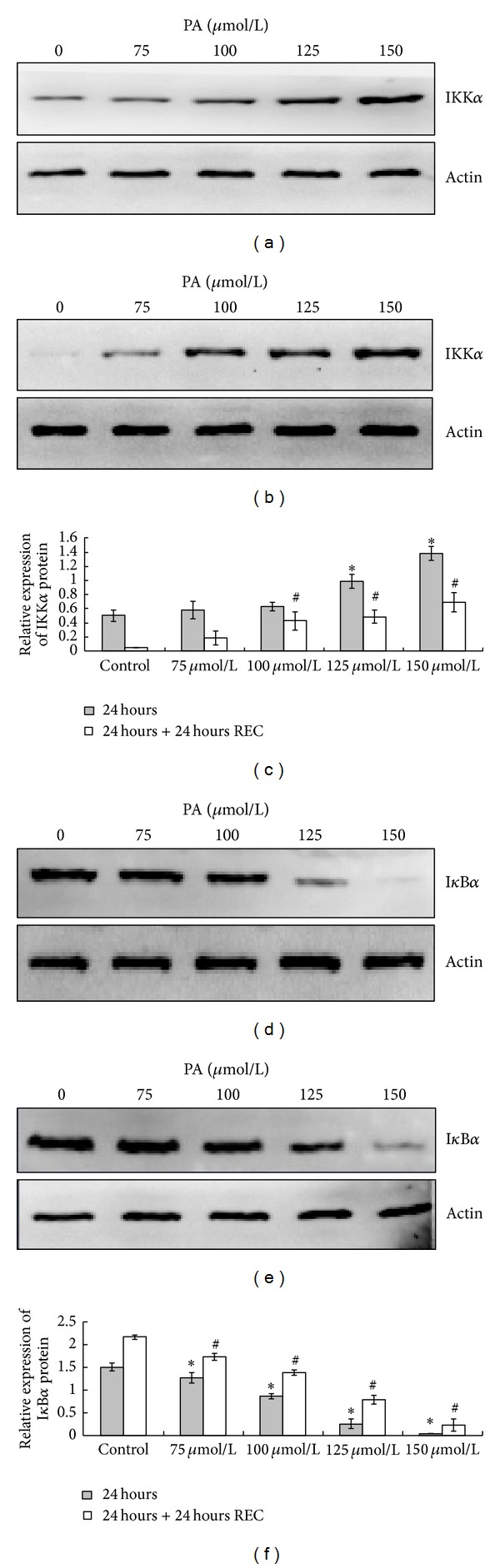
PA induces activation of IKK*α* and degradation of I*κ*B*α* in HaCaT cells measured by western blotting. Keratinocytes were untreated or treated with PA (75, 100, 125, and 150 *μ*mol/L) for 24 hours under serum-free conditions. The cells were harvested, and nuclear extracts were analyzed with IKK*α* by western blot immediately after the treatment (a) and 24 hours after the PA depletion (b). The densitometry values are means ± SD of three independent experiments (c). The protein expression of I*κ*B*α* immediately after the treatment (d) and 24 hours after the PA depletion (e) were also determined by western blot. The densitometry values are means ± SD of three independent experiments (f). *indicate significant differences of *P* < 0.05, respectively, between the PA-treated groups and nontreated group immediately after the treatment groups (24 hours); ^#^indicate significant differences of *P* < 0.05, respectively, between the PA-treated groups and nontreated group in 24 hours after the PA depletion groups (24 hours + 24 hours REC).

Upregulation of IKK*α* protein levels in a dose dependent manner were observed in cells treated with PA at 100, 125, and 150 *μ*mol/L compared to control, immediately after the treatment and 24 hours after the PA depletion (Figures 4(a) and 4(b)). The level of I*κ*B*α* protein expression in 75, 100, 125, and 150 *μ*mol/L PA treated cells decreased dose dependently immediately after the treatment and 24 hours after the PA depletion (Figures 4(d) and 4(e)). The results obtained in these results demonstrate that PA is able to induce NF-*κ*B activation in HaCaT keratinocytes.

### 3.4. Induction of PPAR*α* Expression and Phospho-Stat3 in HaCaT Keratinocytes by PA

**Figure 5:**
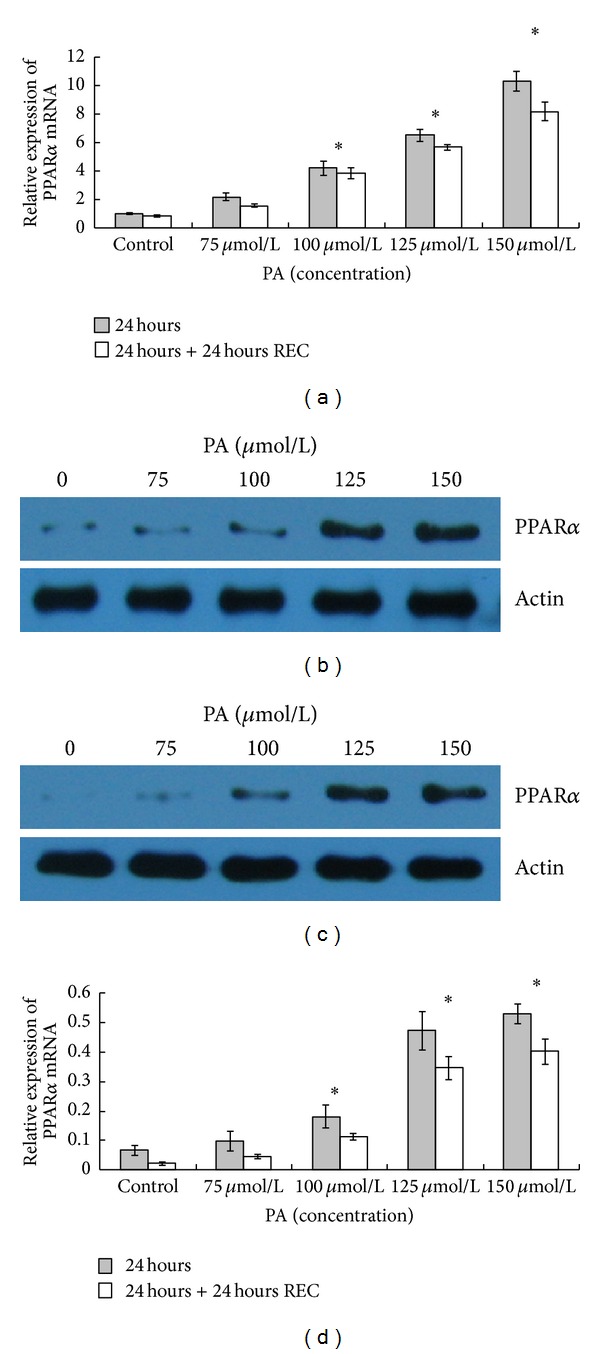
PA induces an upregulation of PPAR*α* mRNA and protein levels in HaCaT keratinocytes. HaCaT keratinocytes were untreated or treated with PA (75, 100, 125, and 150 *μ*mol/L) for 24 hours in serum-free conditions, and collected immediately and 24 hours after PA depletion. (a) PPAR-*α* mRNA levels were determined by real-time RT-PCR. The values shown represent mean ± SD of three experiments. Expression of PPAR*α* after treated or untreated with PA immediately (b) and 24 hours (c) were detected by western blotting. The band intensities were evaluated by densitometric analysis (d). The values shown represent the mean ± SD of three experiments. Asterisks (*) indicate significant differences of *P* < 0.05, respectively, between the PA-treated groups and nontreated group.

**Figure 6:**
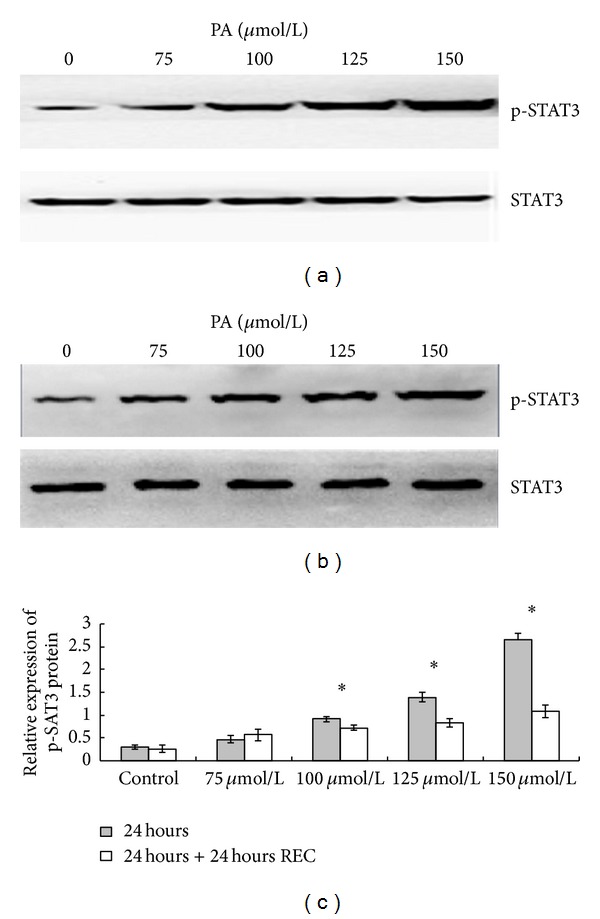
PA induces activation of p-Stat3 (Tyr705) in HaCaT cells measured by western blotting. Keratinocytes were untreated or treated with PA (75, 100, 125, and 150 *μ*mol/L) for 24 hours under serum-free conditions. The cells were harvested, and nuclear extracts were analyzed with p-Stat3 and total Stat3 by western blot immediately after the treatment (a) and 24 hours after the PA depletion (b). The densitometry values are means ± SD of three independent experiments (c). Asterisks (*) indicate significant differences of *P* < 0.05, respectively, between the PA-treated groups and nontreated group.

Immediately after treatment, PA at 75, 100, 125, and 150 *μ*mol/L caused an upregulation of PPAR*α* mRNA levels compared to controls (Figure 5(a)). Afterwards, the level of PPAR*α* gene expression in the treated cells decreased during the following 24 hours (Figure 5(a)). Significant upregulation of PPAR*α* protein levels was observed by means of western blot analysis in cells treated with PA at 100, 125, and 150 *μ*mol/L compared to controls, immediately and 24 hours after PA removal (Figures 5(b) and 5(c)). On the contrary, the treatment of HaCaT keratinocytes with PA at 75 *μ*mol/L did not exert any effect on the expression pattern of PPAR*α*, compared to untreated controls (Figures 5(b) and 5(c)). The PPAR*α* upregulation supports the data mentioned above, which highlighted the possible induction of inflammatory response in HaCaT keratinocytes after the treatment with PA. Significant upregulation of levels of p-Stat3 was also observed by means of western blot analysis in cells treated with PA at 100, 125, and 150 *μ*mol/L compared to total Stat3, immediately and 24 hours after PA removal (Figures 6(a) and 6(b)). 

### 3.5. PA-Induced IL-6, IL-1*β*, and TNF-*α* Production Is Attenuated by Inhibitor of NF-*κ*B

**Figure 7:**
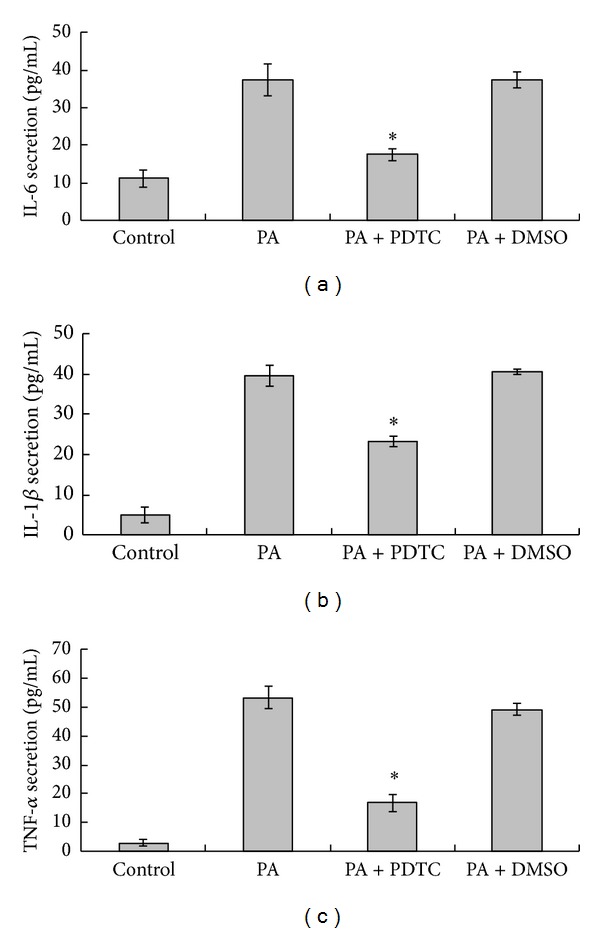
Effect of NF-*κ*B inhibitor PDTC on PA-induced IL-6, IL-1*β*, and TNF-*α* production. HaCaT cells were untreated or exposed to 0.15 mM PA for 24 hours, either without or with pretreatment for 1 hour with 10 *μ*mol/L PDTC or 0.1% dimethylsulfoxide (DMSO, as vehicle control). IL-6 (a), IL-1*β* (b), and TNF-*α* (c) productions were measured by ELISA. Each experiment was done in triplicate. Data are mean ± standard deviation. *P < 0.05 compared with PA.

We examined the effect of inhibition of NF-*κ*B on IL-6, IL-1*β*, and TNF-*α* expression in response to PA. As shown by CCK-8 detection, pretreatment for 1 hour with 10 *μ*M PDTC, a selective chemical inhibitor of NF-*κ*B, had no obvious toxicity to HaCaT cell viability. Moreover, 10 *μ*M PDTC was proved to inhibit PA-induced IKK*α* activation and I*κ*B*α* degradation (see supplementary Figure S1 in Supplementary Material available online at http://dx.doi.org/10.1155/2013/530429). The solvent dimethylsulfoxide (0.1%) was used as a vehicle and control. PA-stimulated IL-6, IL-1*β*, and TNF-*α* productions were significantly attenuated by PDTC, showing that these PA-induced proinflammatory cytokine expressions involve activation of NF-*κ*B activation (Figure 7). Dimethylsulfoxide had no effect on PA-induced IL-6, IL-1*β*, and TNF-*α* production. 

### 3.6. Induction of Cell Proliferation by PA Is Dependent of IL-6 Production

**Figure 8:**
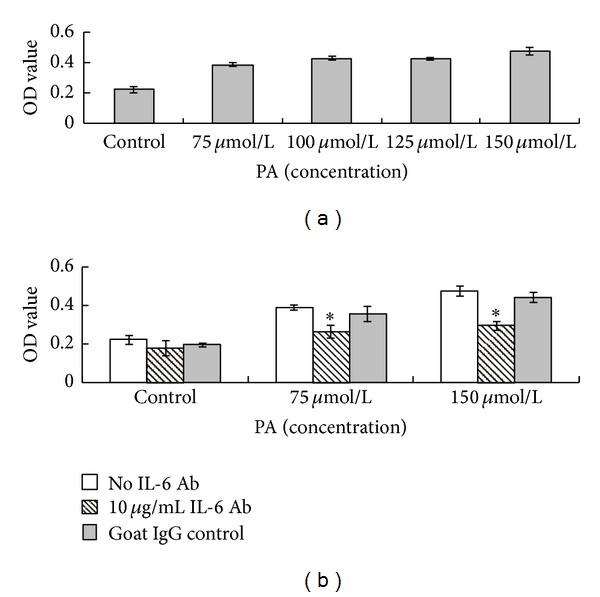
Induction of cell proliferation by palmitic acid is dependent of IL-6. (a) Cells were untreated or treated with PA (75, 100, 125, and 150 *μ*mol/L) for 24 hours. Cell viability was measured by a CCK-8 assay kit. Data are expressed as mean ± standard deviation (*n* = 5). *P < 0.05 compared with control. (b) Cells were untreated or treated with PA (75 and 150 *μ*mol/L) for 24 hours, with or without the presence of 10 *μ*g/mL goat anti-human IL-6 polyclonal neutralizing antibody (IL-6 Ab) or control goat IgG in cell culture system. Cell viability was measured by a CCK-8 assay kit. Data are expressed as mean ± standard deviation (*n* = 5). *P < 0.05 compared with treatments without IL-6 Ab.

In order to evaluate the possible role of PA in the hyperkeratinization of the pilosebaceous duct, we focused on the proliferative response of HaCaT keratinocytes after treatment with PA in a concentration range of 75 to 150 *μ*mol/L by means of CCK-8 test. We observed that PA concentrations between 75 and 150 *μ*mol/L induced a significant proliferative stimulus (Figure 8(a)). These data suggest that PA may be involved in the induction of the hyperkeratosis of the pilosebaceous duct. Interestingly, PA-induced cell proliferative effect was significantly attenuated by the addition of 10 *μ*g/mL goat anti-human IL-6 polyclonal neutralizing antibody in cell culture system (Figure 8(b)). These data demonstrate that autocrine IL-6 production is causally linked to cell proliferation in this in vitro model.

## 4. Discussion

In this study, we supplemented HaCaT cells with PA to mimic the influx of excess FFAs into keratinocytes. For experiments, we used the human keratinocyte cell line HaCaT, which retains biochemical and morphological properties characteristic of keratinocytes, and has proven useful for studying the possible roles of inflammatory mediators in acne vulgaris [25].

Our data demonstrate that exposure of keratinocytes (HaCaT cells) to pathophysiologically relevant concentrations of PA results in increased IL-6, IL-1*β*, and TNF-*α* secretion. This is in agreement with several studies that suggest a link between these three proinflammatory cytokines and PA-induced inflammation [26, 27]. For example, Staiger et al. reported palmitate-induced interleukin-6 expression in human coronary artery endothelial cells and suggested a potential contribution of palmitate to vascular inflammation [27]. Importantly, evidence from previous publications shows that IL-6 is elevated in patients with acne, suggesting that this inflammatory cytokine also may contribute to the development of acne [21]. It was reported that PA significantly decreased H_2_O_2_ generation both by neutrophils and in the xanthine-xanthine oxidase system, while neutrophil chemotaxis and phagocytosis as well as O_2_^−^ and OH^∙^ generation by both systems were not markedly affected in the presence of PA [17]. The authors suggested that PA may be involved in the pathogenesis of acne inflammation from a standpoint of oxidative tissue injury [17]. Besides, it is well documented that IL-6 phosphorylates transcription factor Stat3 at Tyr705 residue and has a role in inflammation [28–30]. Our present data showed that the p-Stat3 level was significantly induced following PA treatment. In this context, we suggested that PA may also contribute to acne inflammation via increasing IL-6 secretion.

Besides IL-6, we also found that TNF-*α* and IL-1*β* were increased following PA treatment, which are also critically important in acne inflammation. These two proinflammatory cytokines not only amplify the NF-*κ*B signaling pathways that originally led to their production through cell surface receptor activation (an autocrine loop), but also will stimulate nearby cells in a paracrine manner. For example, TNF-*α* and IL-1*β* are known to upregulate adhesion molecules, such as ICAM-1 and VCAM-1 on endothelial cells [31, 32]. Thus, the observation that ICAM-1, E-selectin, and VCAM-1 expression levels on the luminal surface of endothelial cells are increased in inflammatory acne papules may be a consequence of TNF-*α* and IL-1*β* induction in the milieu [33]. The elaboration of adhesion molecules is necessary to slow the flow of circulating inflammatory cells for their eventual diapedesis into the inflamed tissue [31, 32].

NF-*κ*B is known to be an important transcription factor for proinflammatory gene expression, and appears to regulate IL-6, IL-1*β*, and TNF-*α* secretions in a cell type-specific and stimulus-specific manner [34, 35]. Activation of NF-*κ*B is mediated through the action of a family of serine/threonine kinases known as I*κ*B kinase (IKK). The IKK (IKK*α* and/or IKK*β*) phosphorylates I*κ*B proteins and the members of the NF-*κ*B family. In our current study, we observed that NF-*κ*B p65 is activated in a dose-dependent manner in human HaCaT cells following PA exposure and subsequently translocated to the nucleus. PA exposure also resulted in an increased degradation of I*κ*B*α* protein. This suggests that the activation of NF-*κ*B p65 in HaCaT cells is mediated through the inhibition of I*κ*B*α* protein proteolysis. It is well documented that I*κ*B*α* is bound to NF-*κ*B p65 through a protein-protein interaction and thus prevents migration of NF-*κ*B p65 into the nucleus [36]. Additionally, the IKK complex is an important site for integrative signals that regulate the NF-*κ*B pathway. We observed that PA exposure resulted in an increase in IKK*α* protein expression in HaCaT cells. These data suggest that PA-induced NF-*κ*B activation and nuclear translocation of NF-*κ*B p65 through greater activation of IKK*α* and degradation of I*κ*B*α* proteins. Besides, PA-induced IL-6, IL-1*β*, and TNF-*α* expressions were attenuated by a NF-*κ*B inhibitor, strongly implicating this transcription factor in the regulation of PA-induced IL-6, IL-1*β*, and TNF-*α* expressions. PPAR*α* exerts a modulatory role in the control of the inflammatory response by antagonizing NF-*κ*B signaling pathway. After the treatment with PA, HaCaT keratinocytes exhibited higher levels of PPAR*α* transcripts probably as a feedback in anti-inflammatory response to the stimulus. 

Our study clearly demonstrates that exposure to excess PA induces HaCaT keratinocyte proliferation and IL-6 production in HaCaT keratinocytes. IL-6 is reported to stimulate keratinocyte proliferation and is therefore studied in diseases associated with epidermal hyperplasia and in wound healing [37–39]. Sebaceous duct keratinocytes from comedones exhibit a hyperproliferative response compared to normal keratinocytes [40, 41]. The hyperproliferative behavior of HaCaT keratinocytes induced by PA could support the idea that FFA may be involved in comedone formation and, in particular, that PA may be responsible for this event. However, whether IL-6 is responsible for the PA-induced HaCaT keratinocyte proliferation is not yet clear. Our data suggested that the PA-induced HaCaT cell proliferation was attenuated by adding IL-6 polyclonal neutralizing antibody into cell culture system, strongly indicating that autocrine of IL-6 is primarily responsible for regulation of PA-induced keratinocytes proliferation. Hence, the interrelation between PA-induced keratinocyte proliferation and IL-6 production may be an important factor in comedone formation. 

In conclusion, we show for the first time that one major type of FFA, palmitic acid, stimulates human HaCaT keratinocytes to produce proinflammatory cytokines IL-6, IL-1*β*, and TNF-*α* in a dose-dependent manner, via activation of NF-*κ*B. Besides, the elevated level of IL-6 may also contribute to PA-induced keratinocyte proliferation. 

## Supplementary Material

Supplementary Figure: Effect of NF-*κ*B inhibitor PDTC on HaCaT cell viability and PA–induced NF-*κ*B activation. (A) HaCaT cells were untreated or exposed to PDTC (10, 50, 100 *μ*mol/L) for 1 hour. Cell viability was measured by a CCK-8 assay kit. Data are expressed as mean ± standard deviation (n = 5). *∗*P < 0.05 compared with control. (B) HaCaT cells were untreated or exposed to 0.15 mM palmitic acid (PA) for 24 hours, either without or with pretreatment for 1 hour with 10 *μ*mol/L PDTC or 0.1% dimethylsulfoxide (DMSO, as vehicle control). IKK*α* and I*κ*B*α* expression levels were measured by western blotting. Each experiment was done in triplicate. Data are mean ± standard deviation. *∗*P < 0.05 compared with PA.

## Figures and Tables

**Table 1:** Primers used in the real-time RT-PCR amplification of the human PPAR-*α* gene and GAPDH mRNAs.

Gene name		primer sequences
PPAR*α*	Forward primer	5′-TTCGCAATCCATCGGCGAG-3′
	Reverse primer	5′-CCACAGGATAAGTCACCGAGG-3′

GAPDH	Forward primer	5′-TGTTGCCATCAATGACCCCTT-3′
	Reverse primer	5′-CTCCACGACGTACTCAGCG-3′
