# Supplementary figures and images for: Palmitic Acid Induces Production of Proinflammatory Cytokines Interleukin-6, Interleukin-1β, and Tumor Necrosis Factor-α via a NF-κB-Dependent Mechanism in HaCaT Keratinocytes
Source: Mediators Inflamm. 2013 Aug 29;2013:530429. doi: 10.1155/2013/530429 (PMC3774064; doi:10.1155/2013/530429)

A

B


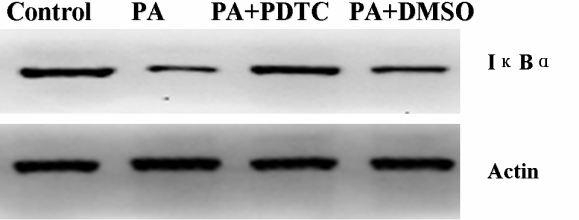

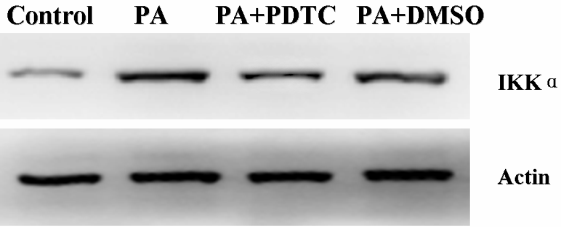

Supplement: Supplementary file 1 — Supplementary Figure: Effect of NF-κB inhibitor PDTC on HaCaT cell viability and PA–induced NF-κB activation. (A) HaCaT cells were untreated or exposed to PDTC (10, 50, 100 μmol/L) for 1 hour. Cell viability was measured by a CCK-8 assay kit. Data are expressed as mean ± standard deviation (n = 5). ∗P < 0.05 compared with control. (B) HaCaT cells were untreated or exposed to 0.15 mM palmitic acid (PA) for 24 hours, either without or with pretreatment for 1 hour with 10 μmol/L PDTC or 0.1% dimethylsulfoxide (DMSO, as vehicle control). IKKα and IκBα expression levels were measured by western blotting. Each experiment was done in triplicate. Data are mean ± standard deviation. ∗P < 0.05 compared with PA. [file 530429.f1.docx]
